# Abnormal Cortico-Cerebellar Functional Connectivity in Autism Spectrum Disorder

**DOI:** 10.3389/fnsys.2018.00074

**Published:** 2019-01-15

**Authors:** Taiane Coelho Ramos, Joana Bisol Balardin, João Ricardo Sato, André Fujita

**Affiliations:** ^1^Department of Computer Science, Institute of Mathematics and Statistics, University of São Paulo, São Paulo, Brazil; ^2^Brain Institute, Hospital Israelita Albert Einstein, São Paulo, Brazil; ^3^Center of Mathematics, Computation, and Cognition, Universidade Federal do ABC, Santo André, Brazil

**Keywords:** cortico-cerebellar connectivity, autism spectrum disorders, ASD, resting-state fMRI, cerebellum, functional connectivity, underconnectivity, ABIDE

## Abstract

The cerebral cortex and the cerebellum are spatially remote areas that are connected by complex circuits that link both primary and associative areas. Previous studies have revealed abnormalities in autism spectrum disorder (ASD); however, it is not clear whether cortico-cerebellar connectivity is differentially manifested in the disorder. To explore this issue, we investigated differences in intrinsic cortico-cerebellar functional connectivity between individuals with typical development (TD) and those with ASD. To this end, we used functional magnetic resonance imaging (fMRI) of 708 subjects under a resting state protocol provided by the ABIDE I Consortium. We found that people with ASD had diminished functional connectivity between the cerebellum and the following cortical regions: (i) right fusiform gyrus, (ii) right postcentral gyrus, (iii) right superior temporal gyrus, (iv) right middle temporal gyrus, and (v) left middle temporal gyrus. All of these regions are involved in many cognitive systems that contribute to commonly affected functions in ASD. For right fusiform gyrus, right superior temporal gyrus, and left middle temporal gyrus, we reproduced the results in an independent cohort composed of 585 subjects of the ABIDE II Consortium. Our results points toward a consistent atypical cortico-cerebellar connectivity in ASD.

## 1. Introduction

Autism spectrum disorders (ASD) are mainly characterized by repetitive behavior and social impairment, including differentiated sensitivity to sound and touch and difficulty in recognizing non-verbal language and facial expressions (American Psychiatric Association, 2013). These symptoms may affect a child's cognitive development, which may prevent self-sufficiency in adulthood. One out of 68 children in the U.S. (Christensen, 2016) and 1% of the population worldwide (Elsabbagh et al., 2012) are estimated to have ASD. Despite this high incidence, the pathophysiology of ASD is still unclear. Therefore, additional studies are needed to better understand the underlying mechanisms and develop proper treatments for ASD.

Cerebellar abnormalities have been implicated in ASD (Fatemi et al., 2012), as suggested by studies correlating ASD to a reduced number and size of Purkinje cells (Fatemi et al., 2002; Bauman and Kemper, 2005) and cerebellar vermis hypoplasia (Courchesne et al., 1988; Hashimoto et al., 1995; Webb et al., 2009). Moreover, cerebellar lesions in premature children may result in symptoms similar to ASD (Limperopoulos et al., 2014). Although the cerebellum was originally considered a motor structure, (Schmahmann, 2010; Noroozian, 2014; Baumann et al., 2015; Hoche et al., 2016) identified its role in cognitive, social, and emotional abilities, in special by observations of the cerebellar cognitive affective syndrome (Schmahmann, 2004). Its involvement in these functions may be explained by the vast white matter pathways connecting the cerebellum to functionally heterogeneous cortical regions (Glickstein, 1992; Ramnani, 2006). Functional cortical-cerebellar connectivity studies suggest a correlation between the cerebellar activity and several cortical regions, particularly correlation between the cerebellar frontal lobe and temporal, auditory, superior temporal, somatosensory, motor, and premotor regions and between the cerebellar posterior lobe and posterior parietal regions and the prefrontal cortex (O'Reilly et al., 2010; Buckner et al., 2011). In addition, meta-studies report cerebellar activity related to higher cognitive domains, including language, verbal working memory, and emotional processing (Stoodley and Schmahmann, 2009; Buckner, 2013; Keren-Happuch et al., 2014).

Currently, ASD is believed to be a disorder related more to differential brain connectivity than to the activity in a specific brain region (Müller et al., 2011; Maximo et al., 2014). Thus, it is natural to ask whether cortico-cerebellar connections are differentiated in ASD compared to typical development (TD). Attempts to answer this question are usually based on diffusion imaging studies, most of which show decreased Fractional Anisotropy (FA) suggesting a weaker structural connectivity in participants with ASD (Catani et al., 2008; Brito et al., 2009; Hanaie et al., 2013) (for a review, see Crippa et al., 2016). Task-driven fMRI studies reported decreased functional cortico-cerebellar connectivity during finger tapping task (Mostofsky et al., 2009) and verb generation task (Verly et al., 2014) in children with ASD.

A potential technique for studying neural connectivity is resting-state fMRI (rs-fMRI). rs-fMRI measures fluctuations in the blood oxygen level-dependent (BOLD) signal when a subject is not performing any specific task (Biswal et al., 1995). The brain areas and their respective correlations between the BOLD signals form the functional network (Fox and Raichle, 2007), which has been shown to be a good approximation of structural connections (Smith et al., 2009). However, few rs-fMRI studies focus specifically on cortico-cerebellar connectivity alterations in ASD. To the best of our knowledge, the study by Khan et al. (2015) is the only one that aimed at assessing functional cortico-cerebellar connectivity using rs-fMRI. The authors found a general cortico-cerebellar overconnectivity in children and adolescents with ASD, in special in sensori-motor networks, accompanied by underconnectivity in supramodal networks. They relate these results with previous findings of early overgrowth of the white matter, possibly leading to poorly assembled networks.

Considering the evidence, the current study aimed to identify brain regions with differential functional connectivity with the cerebellum in ASD, using rs-fMRI in a public large discovery sample (ABIDE I dataset) and to validate the findings in a validation sample (ABIDE II dataset). To the best of our knowledge, this is the first work to assess functional cortico-cerebellar connectivity that reproduced results on an independent dataset.

## 2. Materials and Methods

To test our hypothesis of differential cortico-cerebellar functional connectivity between subjects with TD and those with ASD, we downloaded a large fMRI dataset from the ABIDE I Consortium and confirmed the results using an independent dataset (ABIDE II).

### 2.1. Functional MRI Data

We downloaded two large resting-state fMRI datasets, namely, ABIDE I and ABIDE II. ABIDE I is composed of 573 individuals with TD and 539 individuals with ASD (totaling 1 112 subjects). ABIDE II is composed of 593 individuals with TD and 521 individuals with ASD (totaling 1 114 subjects). After preprocessing (described in section 2.2.), the ABIDE I dataset was composed of 432 subjects with TD (348 males, mean age ± standard deviation of 18.21±8.04) and 276 individuals with ASD (241 males, 18.42±8.38). The ABIDE II dataset was composed of 316 subjects with TD (208 males, 12.80±5.61) and 269 individuals with ASD (234 males, 13.93±7.07). Both are available on the ABIDE Consortium website (http://fcon_1000.projects.nitrc.org/indi/abide/). According to the ABIDE repository, the acquisition methods and protocols were approved by the corresponding local Institutional Review Boards (i.e., the review boards and their regulations at the California Institute of Technology, Carnegie Mellon University, ETH Zürick, Georgetown University, Indiana University, Kennedy Krieger Institute, University of Leuven, Ludwig Maximilians University Munich, New York University, Oregon Health and Science University, Institute of Living at Hartford Hospital, University of Pittsburgh, Social Brain Lab, San Diego State University, Stanford University, Trinity Center for Health Sciences, University of California Davis, University of California Los Angeles, University of Michigan, University of Utah School of Medicine, Yale School of Medicine) and were performed in accordance with Health Insurance Portability and Accountability Act (HIPAA) guidelines and the 1,000 Functional Connectomes Project/International Data-sharing Initiative (http://fcon_1000.projects.nitrc.org/) protocols. Written informed consent was obtained from all the participants. All data distributed via the ABIDE website were fully anonymized in compliance with the HIPAA privacy rules, and no protected health information was included. The imaging protocols are considered to be equivalent across different institutes. Further details about this dataset can be obtained from the ABIDE consortium website.

### 2.2. Image Preprocessing

We preprocessed the imaging data using the Athena pipeline (www.nitrc.org/plugins/mwiki/index.php/neurobureau:AthenaPipeline). The pipeline focused on providing systematic processing of fMRI data, including the following main steps: exclusion of the first four scans; slice timing correction; deoblique dataset; correction for head movements; masking the volumes to exclude non-brain regions; co- registration of the mean image to the respective anatomic image of the subject; spatial normalization to MNI space (4 × 4 × 4 mm resolution); extraction of BOLD (Ogawa et al., 1990) time series from white matter (WM) and cerebrospinal-fluid (CSF); removing the effects of WM, CSF, motion, and trend using multiple linear regression; temporal band-pass filter (0.009 < *f* < 0.08 Hz); and spatially smoothing the filtered data using a Gaussian filter (FWHM = 6 mm). All these steps were performed by using the following software: Analysis of Functional NeuroImages (AFNI) (http://afni.nimh.nih.gov/afni) and the fMRIB Software Library (FSL) (http://fsl.fMRIb.ox.ac.uk/fsl/fslwiki/). To define the 116 ROIs considered in this study, we used the Automated Anatomical Labeling (AAL) atlas (Tzourio-Mazoyer et al., 2002). We identified 25 ROIs comprising the ventricles by using the Montreal Neurological Institute (MNI) atlas, and we removed them. The head coil coverage available in the scanners may vary depending on the model, which may lead to a lack of reading of part of the cerebellum for some subjects. Thus, to avoid artifacts, we adopted a masking procedure to constrain the statistics only to ROIs with a mean voxels sampling of 80% across participants. An image was created from the overlay plot to represent the percentage of valid voxels in each cerebellar ROI (see Figure S1). We found that ROIs from I-VI lobes and the vermis region matched this criterion. Thus, we considered 79 ROIs (65 cortical and 14 cerebellar) for further analysis. Subjects' head movements during MRI scanning could lead to spurious correlations between ROIs; thus, we carried out the “scrubbing” procedure (Power et al., 2012) to remove frames affected by head movement. We removed frames that presented both framewise displacement (FD) greater than 0.5 mm and DVARS greater than 0.5% ΔBOLD (Power et al., 2012). Preceding and following frames that did not meet these criteria were not removed. Subjects that had more than 5% of the total number of scans removed by scrubbing were excluded from the analysis. Head motion across the dataset is measured by the FD, which presents a mean of 0.137 mm and standard deviation of 0.102 mm on the study dataset (ABIDE I) and a mean of 0.169 mm with standard deviation of 0.132 mm on the independent dataset (ABIDE II). A total of 93 subjects (53 TD and 40 ASD) in the study dataset (ABIDE I) and 45 subjects (24 TD and 21 ASD) in the independent dataset (ABIDE II) had scans removed by the scrubbing process.

We considered both autism and Asperger syndrome as parts of the ASD as suggested by the Diagnostic and Statistical Manual of Mental Disorders 5th edition (DSM-5) (American Psychiatric Association, 2013).

### 2.3. Cortico-Cerebellar Functional Connectivity Analysis

To summarize, reduce the number of variables, and enhance the statistical power, for each subject in the sample, we applied the principal component analysis (PCA) on the 14 cerebellar ROI time series. Then, for each subject, we selected the principal components (PCs) that explained at least 95% of the data variance. Thus, each subject had a different number of PCs used for the analyses (average of 9.25 PCs with standard deviation of 1.19).

Next, to identify the cortical regions that are functionally associated with the cerebellum, for each subject, we carried out a linear regression with the cortical ROI time series as response variables and the PCs of the cerebellum obtained by the PCA for the subject as predictor variables. The adjusted *R*^2^ (coefficient of determination) was considered as a measure of functional connectivity between the cortical ROI and the cerebellum.

To test if the functional connectivity was different between the TD and ASD groups for each cortical ROI, we performed a linear regression using the measure of functional connectivity (the adjusted *R*^2^ obtained for each subject in the previous step) as the response variable and the diagnostic group (TD or ASD) as the predictor variable. To reduce age, gender, and site effects, we included them as covariates in this linear model. All p-values were corrected for multiple tests by using the False Discovery Rate (FDR) (Benjamini and Hochberg, 1995) approach (we considered all 65 tests, one for each cortical ROI). The corrected *p*-value threshold considered to be statistically significant was set at 5%.

## 3. Results

**Figure 1:**
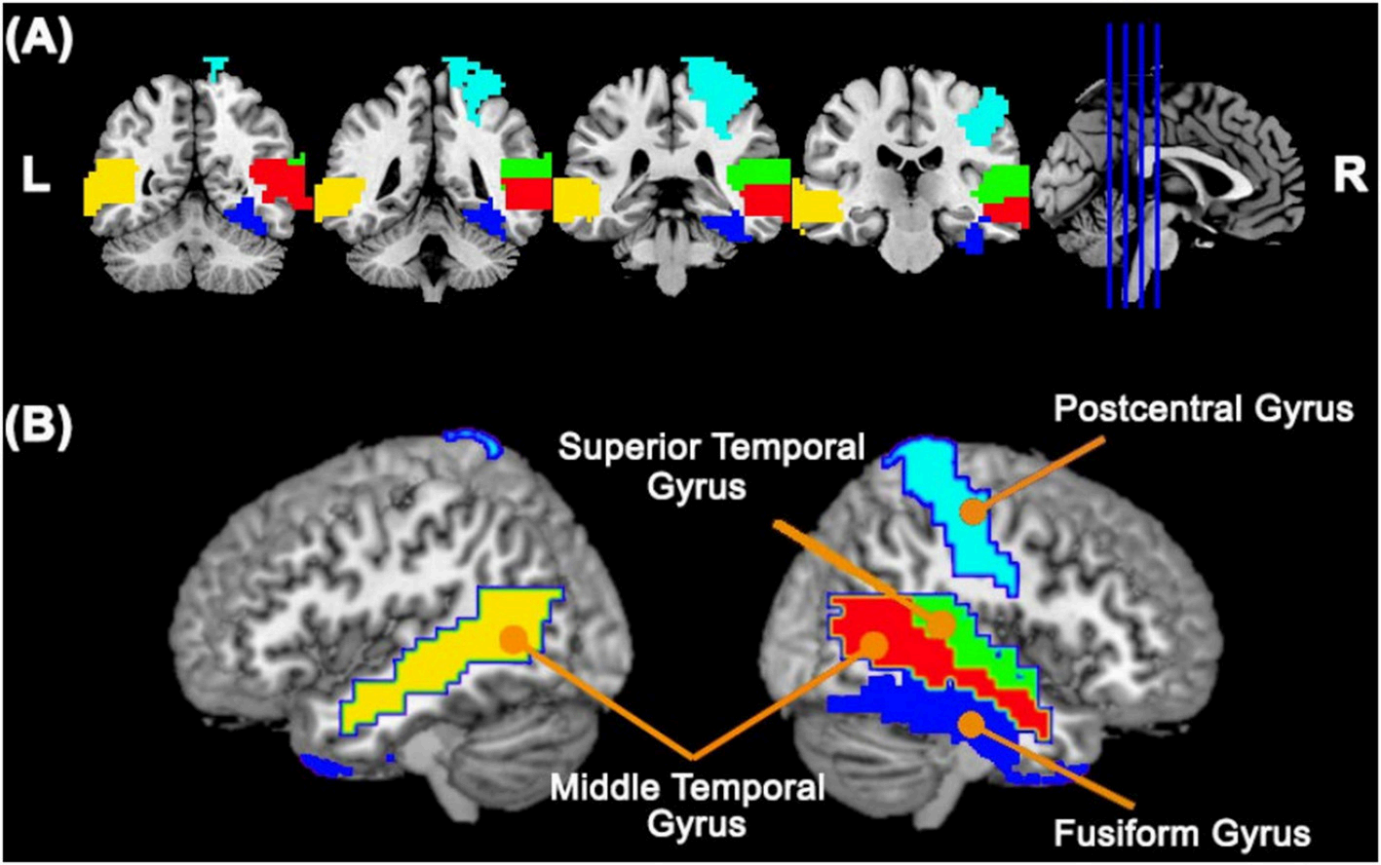
Cortical ROIs with a differential association with the cerebellum between TD and ASD groups obtained by analyzing the ABIDE I dataset. Panels **(A,B)** represent coronal slices and two lateral views of the brain, respectively. The colors represent the cortical ROIs that differentially associated with the cerebellum between TD and ASD groups, namely, the right fusiform gyrus, right postcentral gyrus, right superior temporal gyrus, right middle temporal gyrus, and left middle temporal gyrus.

**Figure 2:**
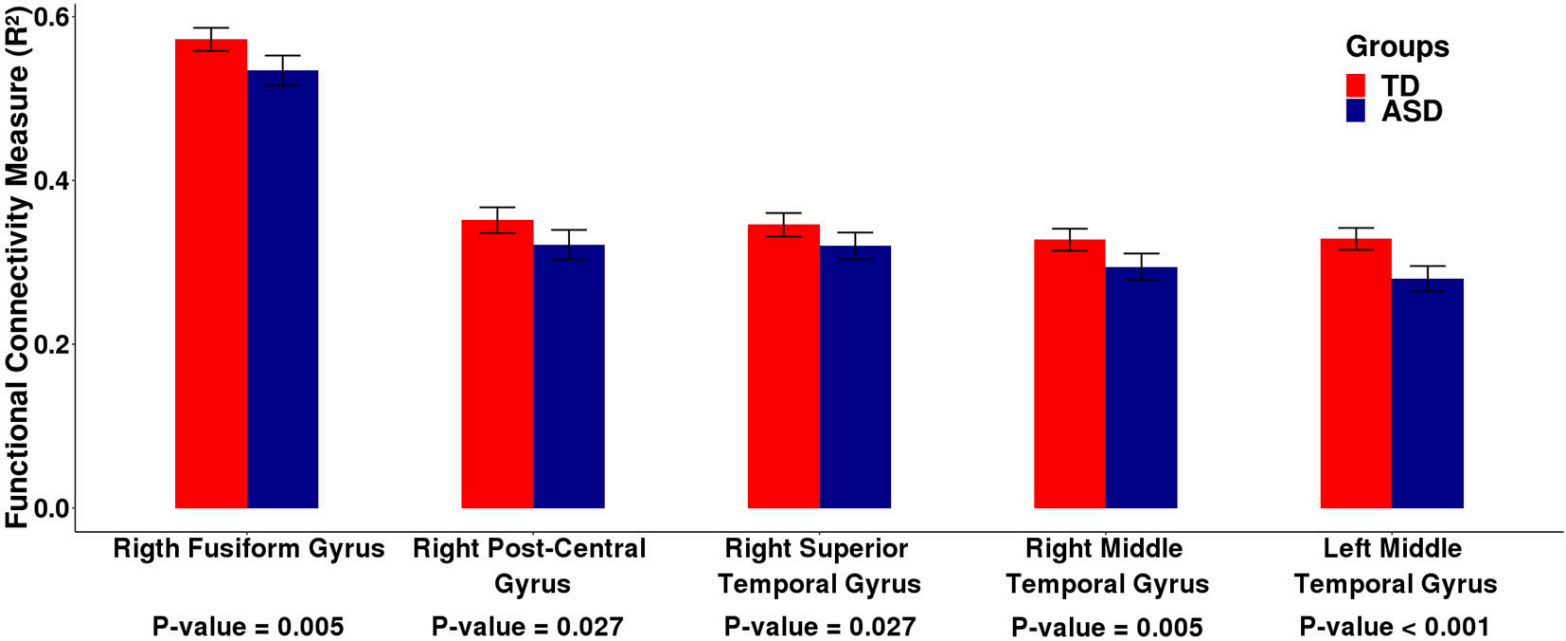
Barplots of the functional connectivity measure (*R*^2^) between the cortical ROI and the cerebellum obtained by analyzing the ABIDE I dataset. Each pair of bars represent the mean functional connectivity measurements between the cortical ROI and the cerebellum for the TD and ASD groups, respectively. The error bars represent 95% confidence interval. *P*-values were obtained by linear regression with the measure of functional connectivity (*R*^2^) as the response variable and the diagnostic group (TD or ASD) as the predictor variable with age, gender, and site as covariates. All *p*-values were corrected for multiple tests by FDR. Note that the mean functional connectivity measurement (*R*^2^) is significantly lower in the ASD group compared to the TD group, suggesting decreased functional cortico-cerebellar connectivity in the ASD group.

First, we carried out the procedures described in the sections 2.2. and 2.3. using the ABIDE I dataset. In summary, for each subject, we carried out a linear regression between the fMRI time series of each cortical ROI and the cerebellum. To represent the neural activity of the cerebellum, we used the cerebellar PCs representing 95% of the variance of the time series in this region. We estimated the *R*^2^ value of this regression as a measure of connectivity between each cortical ROI and the cerebellum as a whole. Then, we carried out a second linear regression between the *R*^2^ values obtained in the previous linear regression and the group (TD or ASD), by including age, gender, and site as covariates. We identified five cortical ROIs (Figure 1) that are differentially associated with the cerebellum between the typical development (TD) and autism spectrum disorder (ASD) groups, namely, the right fusiform gyrus [β = −0.042, *t*-value = −3.689, *p* = 0.005 —*t*-test (GLM)], the right postcentral gyrus [β = −0.038, *t*-value = −3.131, *p* = 0.027 — t-test (GLM)], the right superior temporal gyrus [β = −0.034, *t*-value = −3.082, *p* = 0.027 –*t*-test (GLM)], the right middle temporal gyrus [β = −0.039, *t*-value = −3.783, *p* < 0.001 —*t*-test (GLM)], and the left middle temporal gyrus [β = −0.056, *t*-value = −5.481, *p* = 0.005 —*t*-test (GLM)]. All p-values were corrected for FDR. Figure 2 presents the barplots for the cortico-cerebellar functional connectivity measurements (adjusted *R*^2^) for the TD and ASD groups for each of the five cortical ROIs. It is important to mention that for all of the identified cortical regions, the cortico-cerebellar functional connectivity in the ASD group was statistically lower than that in the TD group.

**Figure 3:**
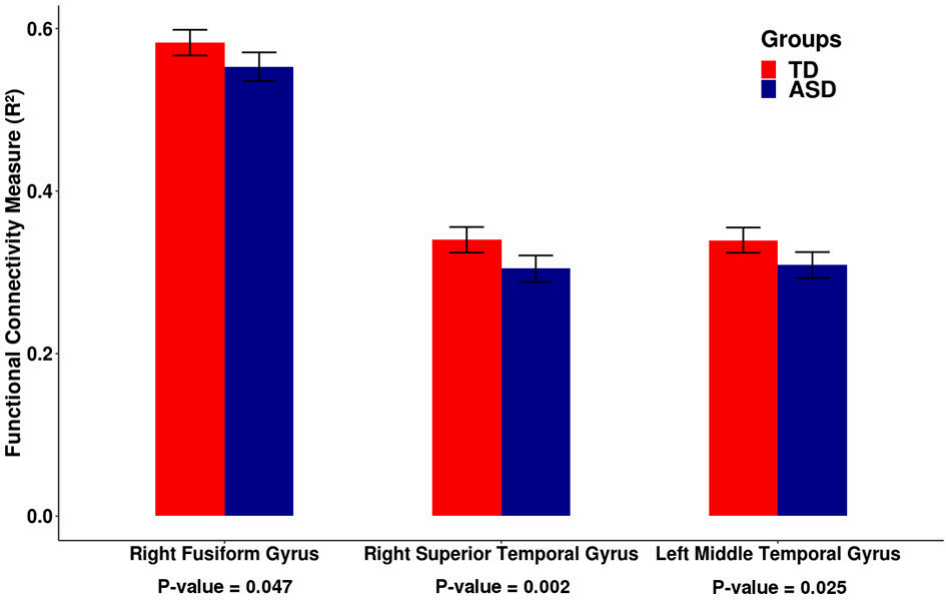
Barplots of the functional connectivity measure (*R*^2^) between the cortical ROI and the cerebellum for an independent dataset (ABIDE II). Each pair of bars represent the mean functional connectivity measurements between the cortical ROI and the cerebellum for the TD and ASD groups, respectively. The error bars represent 95% confidence interval. *P*-values were obtained by linear regression with the measure of functional connectivity (*R*^2^) as the response variable and the diagnostic group (TD or ASD) as the predictor variable with age, gender, and site as covariates. All *p*-values were corrected for multiple tests by FDR. Note that the median functional connectivity measurement (*R*^2^) is significantly lower in the ASD group compared to the TD group, suggesting decreased functional cortico-cerebellar connectivity in the ASD group. These data confirm the findings that were obtained by analyzing the ABIDE I dataset.

Then, to check the reproducibility of the findings, we tested these five differential cortico-cerebellar connectivity areas on an independent dataset composed of 585 subjects collected from 11 sites available in the ABIDE II consortium. The criteria for selecting the participants and the preprocessing procedure adopted for the independent dataset were the same as described for the ABIDE I dataset in the section 2.2. In this validation analysis, three out of five cortical ROIs, namely, the right fusiform gyrus [β = −0.028, *t*-value = −2.193, *p* = 0.047 —*t*-test (GLM)], the right superior temporal gyrus [β = −0.044, *t*-value = −3.495, *p* = 0.002 —*t*-test (GLM)], and the left middle temporal gyrus [β = −0.033, *t*-value = −2.583, *p* = 0.025—*t*-test (GLM)], confirmed the reduced functional connectivity in the ASD group (*p*-values corrected for multiple tests by FDR). The barplots for the functional connectivity measures (adjusted *R*^2^) of these three cortical regions are shown in Figure 3. It is important to mention that the cortico-cerebellar functional connectivity in the ASD group was statistically lower than that in the TD group, as observed in the previous dataset. For some brief results on difference in intracortical connectivity between groups, see Figures S2, S3.

## 4. Discussion

In this study, we investigated the differences in the intrinsic cortico-cerebellar functional connectivity between individuals with TD and those with ASD. We found that people with ASD had diminished functional connectivity between the cerebellum and several cortical regions, including the right postcentral, middle temporal, superior temporal, and fusiform gyri and the left middle temporal gyrus. For the right fusiform gyrus, the right superior temporal gyrus, and the left middle temporal gyrus, the validity of the results was confirmed in a large independent sample (ABIDE II).

Our results are in line with previous studies based on diffusion tensor imaging (DTI) suggesting alterations in structural connectivity in ASD. These studies show reduced values of fractional anisotropy (FA) in the white matter of individuals with ASD (Shukla et al., 2010; Libero et al., 2016) and abnormal development of such white matter pathways in young children on the spectrum (Ben Bashat et al., 2007; Wolff et al., 2012). In addition, there are studies showing specifically reduced FA in the temporal lobe (Barnea-Goraly et al., 2004; Lee et al., 2007), in agreement with our results of reduced functional connectivity in the right middle and superior temporal gyri and left middle temporal gyrus.

Abnormal functional connectivity in the cerebellum has been reported in ASD, albeit with mixed results. For example, cortico-cerebellar functional overconnectivity in the sensorimotor regions and underconnectivity in the supramodal regions were previously described in children and adolescents with ASD (Khan et al., 2015). In adolescents with ASD, reduced connectivity was also shown in the right crus I and several contralateral cerebral regions, including the superior frontal gyrus, middle frontal gyrus, thalamus, and anterior cingulate gyrus, as well as in the precentral gyrus (Verly et al., 2014). In our replication study, a prominent pattern of reduced connectivity in ASD was observed in the lateral temporal cortex. This variation among the results in the different studies may reflect differential alterations in connectivity related to patient characteristics (i.e., age, disease duration, and symptom severity) or image analysis methods, as previously discussed in the autism literature (Ecker et al., 2015). Although the nature and direction of functional connectivity differences in ASD remain inconclusive, our results add to the evidence indicating decreased cortico-cerebellar functional connectivity in patients.

The specific cortical regions that exhibited ASD-related altered functional connectivity with the cerebellum have also been linked to symptoms and traits of autism. For example, differences in frontocerebellar circuits have been associated with motor impairments and stereotyped repetitive behaviors (Floris et al., 2016), and the lateral temporal cortex and fusiform gyrus appear to mediate social processing deficits (for a review, see Just et al., 2012). Altogether, these findings suggest that differences in cortico-cerebellar functional connectivity in organized somatomotor and associative networks may contribute to the clinical manifestations in ASD, although further studies are needed to establish a direct association between our findings and autistic symptoms.

The neurobiological mechanisms underlying the diminished cortico-cerebellar functional connectivity in ASD observed in our study remain speculative. The communication of the cerebellum with the cerebral cortex has been associated with a closed-loop system in which the cerebellum returns projections to the cerebral cortex via the thalamus (for a review, see Ramnani, 2006). In this context, cortico-cerebellar connectivity differences could emerge from developmental alterations in both thalamo-cortical and/or extrinsic cortico-cortical projections. In fact, prior ASD studies using diffusion tensor MRI have detected differences in microstructural integrity of tracts connecting thalamus with motor and somatosensory cortices (Nair et al., 2013), and also of the corpus callosum (Alexander et al., 2007). It is important to note, however, that microstructural alterations have also been reported in cerebellar tracts (e.g., intracerebellar fibers and right superior cerebellar output peduncle) (Catani et al., 2008). Thus, the extent to which the observed functional differences relates to anatomy is unknown and requires investigation.

Moreover, the challenges in the reproducibility of neuroimaging findings (Griffanti et al., 2016) and the influence of small sample size in the reliability of results (Button et al., 2013) have been much discussed in the current scientific literature. A major strength of this study is the reproducibility of most of the results in a large sample of similar individuals from the ABIDE II dataset (Di Martino et al., 2017). On the other hand, there are limitations that need to be taken into consideration. The estimation of cerebellar zones coupled to cerebral regions was compromised by the fact that there was no full coverage of the cerebellum in most individuals, particularly in the posterior region. Therefore, further studies are required to comprehensively examine ASD-related differences in cortico-cerebellar functional connectivity. Importantly, both the ABIDE I and ABIDE II datasets are multicentric, with heterogeneous acquisition parameters across sites. Thus, to minimize the site effect in our analysis, we included it as a covariate in group-level statistics as described in the section 2.3. It is also important to highlight that since the ROIs were divided according to anatomical regions, they could exhibit functional heterogeneity, possibly leading to ambiguous functional connectivity.

In conclusion, our results suggest that ASD displays atypical reduced intrinsic functional cortico-cerebellar connectivity in specific networks, which is consistent with the idea that ASD is a disorder characterized by abnormalities in neural connections (Hoppenbrouwers et al., 2014).

## Author Contributions

TR and AF conceived the analyses. TR conducted the analyses. TR, JB, JS, and AF analyzed the results and reviewed the manuscript. All authors read and approved the final version of the manuscript.

### Conflict of Interest Statement

The authors declare that the research was conducted in the absence of any commercial or financial relationships that could be construed as a potential conflict of interest.
