# Supplementary material for: Abnormal Cortico-Cerebellar Functional Connectivity in Autism Spectrum Disorder
Source: Front Syst Neurosci. 2019 Jan 15;12:74. doi: 10.3389/fnsys.2018.00074 (PMC6341229; doi:10.3389/fnsys.2018.00074)
Supplement: Supplementary file 1 [file Data_Sheet_1.PDF]

Supplementary Material:
Abnormal Cortico-Cerebellar Functional Connectivity in Autism Spectrum Disorder

1 SUPPLEMENTARY FIGURES

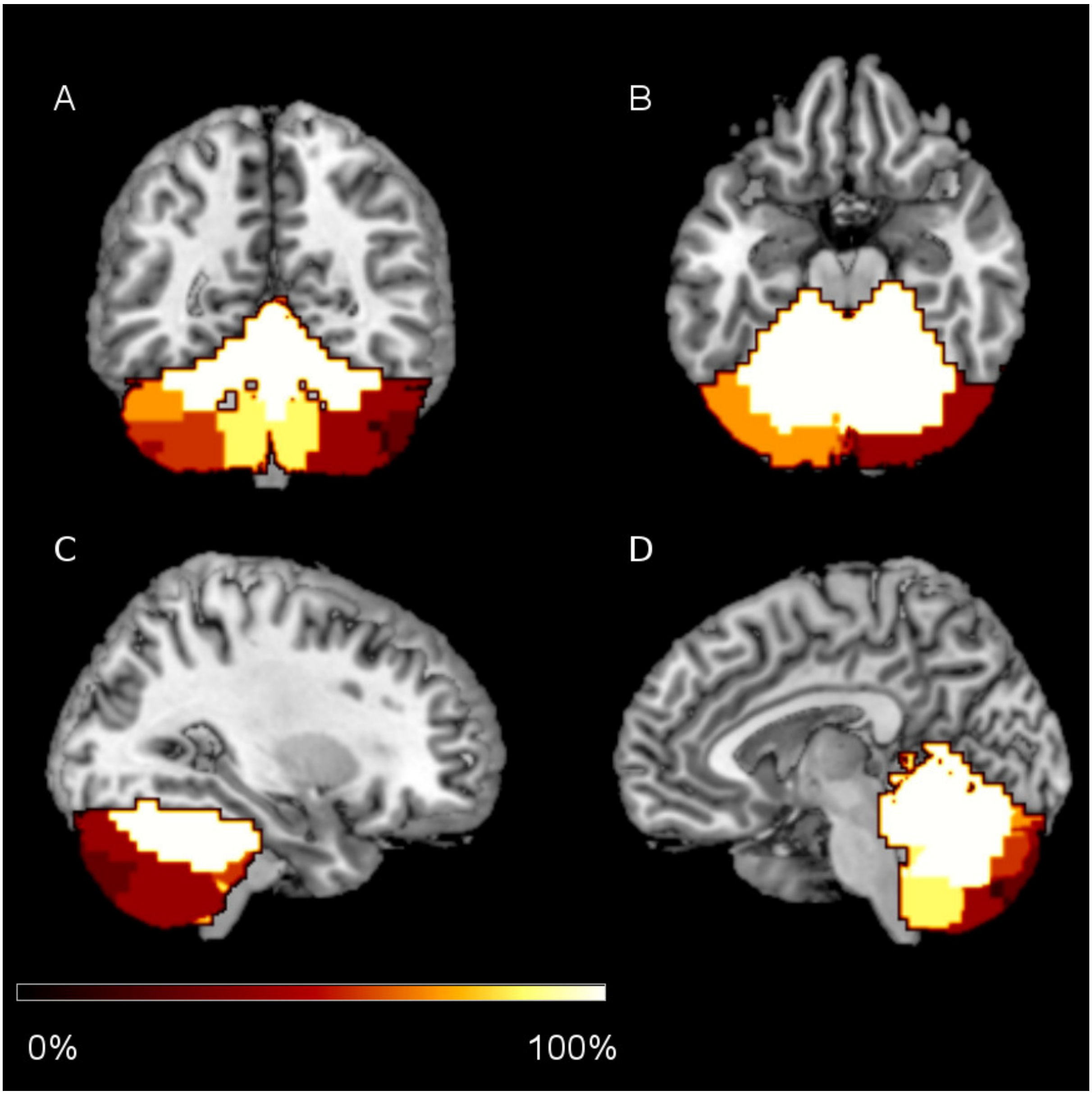

Figure S1. **(A)** Coronal view of the cerebellum. **(B)** Axial view of the cerebellum. **(C)** Sagittal view of the right hemisphere. **(D)** Sagittal view of the vermis. The heatmap was created using the mean voxels sampling of each ROI across participants. Brighter regions are the ROIs considered in our analysis, i.e., with more than 80% of voxels sampled on average, namely ROIs from I-VI lobes and the vermis region.

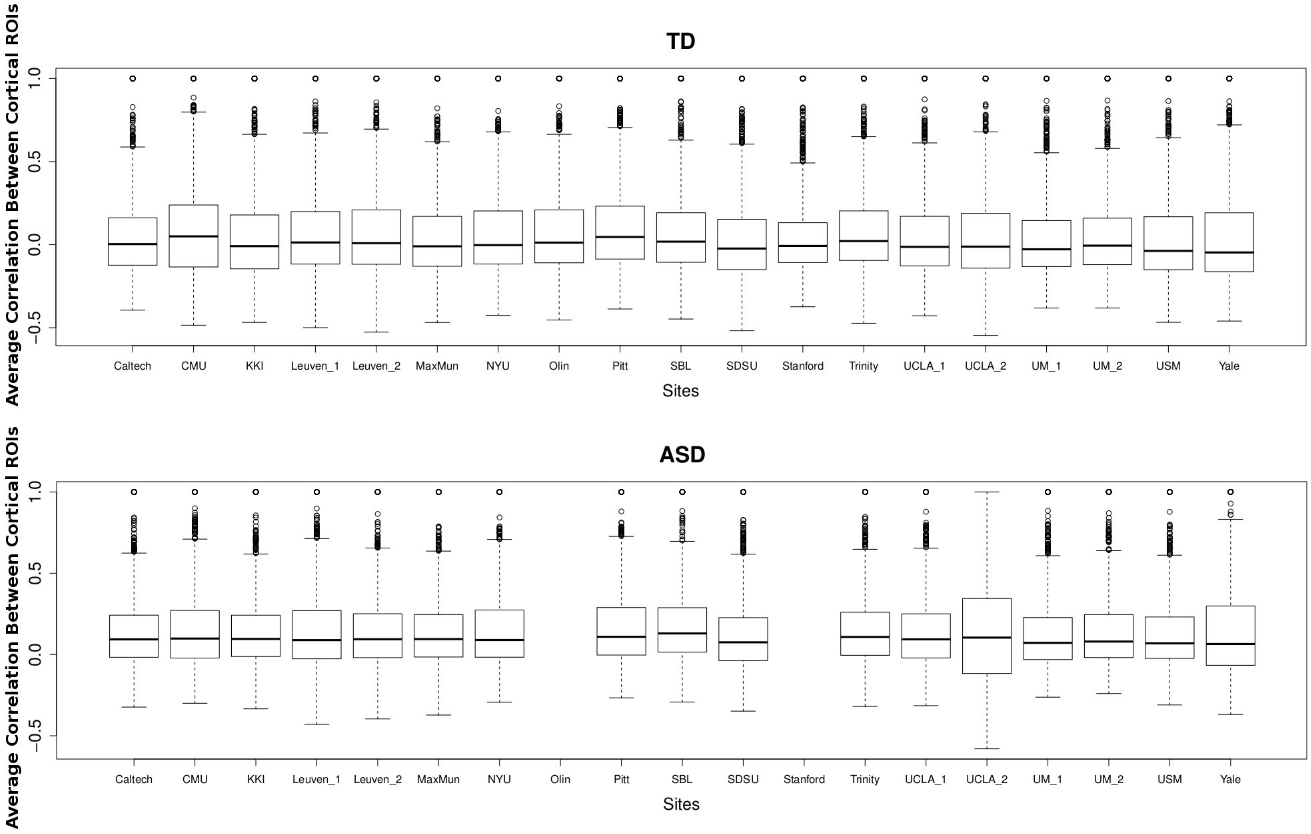

Figure S2. To obtain an intracortical connectivity measure, for each individual we estimate the average Pearson correlation coefficient between all pairs of ROIs in the cortex. Note that the distribution of the average correlation between cortical ROIs is similar across sites. Observation: Olin and Stanford sites contain only TD subjects.

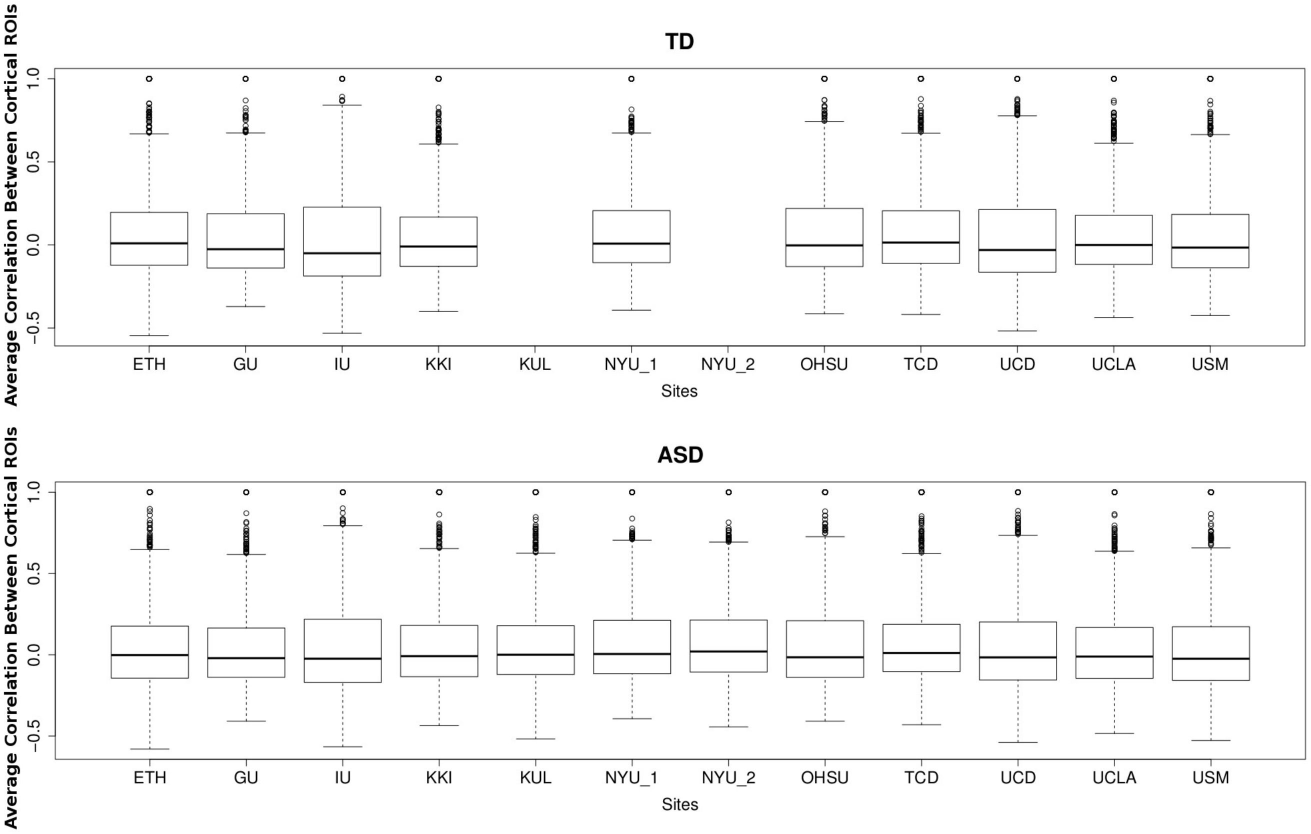

Figure S3. We repeated the same analysis described in Figure S2 for ABIDE II data set. Note that the distribution of the average correlation between cortical ROIs is similar across sites. Observation: KUL and NYU_2 sites contain only ASD subjects.
